# Hybrid Mesh for Nasal Airflow Studies

**DOI:** 10.1155/2013/727362

**Published:** 2013-08-05

**Authors:** Mohammed Zubair, Mohammed Zulkifly Abdullah, Kamarul Arifin Ahmad

**Affiliations:** ^1^School of Aerospace and Mechanical Engineering, Universiti Sains Malaysia, 14300 Nibong Tebal, Pulau Pinang, Malaysia; ^2^Department of Aerospace Engineering, Universiti Putra Malaysia, 43400 Serdang, Selangor, Malaysia

## Abstract

The accuracy of the numerical result is closely related to mesh density as well as its distribution. Mesh plays a very significant role in the outcome of numerical simulation. Many nasal airflow studies have employed unstructured mesh and more recently hybrid mesh scheme has been utilized considering the complexity of anatomical architecture. The objective of this study is to compare the results of hybrid mesh with unstructured mesh and study its effect on the flow parameters inside the nasal cavity. A three-dimensional nasal cavity model is reconstructed based on computed tomographic images of a healthy Malaysian adult nose. Navier-Stokes equation for steady airflow is solved numerically to examine inspiratory nasal flow. The pressure drop obtained using the unstructured computational grid is about 22.6 Pa for a flow rate of 20 L/min, whereas the hybrid mesh resulted in 17.8 Pa for the same flow rate. The maximum velocity obtained at the nasal valve using unstructured grid is 4.18 m/s and that with hybrid mesh is around 4.76 m/s. Hybrid mesh reported lower grid convergence index (GCI) than the unstructured mesh. Significant differences between unstructured mesh and hybrid mesh are determined highlighting the usefulness of hybrid mesh for nasal airflow studies.

## 1. Introduction

The anatomy of the human nasal cavity is further complicated by prevalence of anomalies and diseases. A number of researchers have used computational fluid dynamics (CFD) to study the physiology and fluid flow properties inside the nasal cavity [1–5]. A recent review on the use of CFD for drug delivery design process discussed the importance of using CFD in drug delivery in nasal cavities [6]. However, the accuracy of the CFD study depends primarily on quality and quantity of the mesh distribution. A good mesh must be able to resolve the velocity vectors and effectively capture the fluid properties at all regions inside the nasal cavity. Structured mesh, in spite of its effectiveness in resolving flow properties, is very difficult to develop inside a complicated domain like the nose. Structured mesh was employed only to model the human upper bronchial tree like the trachea and bronchii [7, 8]. Recently, Vinchurkar and Longest [9] considered the effects of various common mesh styles on grid convergence, velocity fields, and particle deposition profiles in a bifurcating respiratory model. The mesh considered included a structured multiblock hexahedral style, an unstructured tetrahedral mesh, a flow-adaptive tetrahedral design, and a hybrid style consisting of tetrahedral and prism elements. However, the work was limited to a small bifurcating section and did not address the entire upper airway domain. It is easy to develop structured mesh in a simple bifurcating domain. But when it comes to the complicated nasal cavity, the process is very tedious and prohibitive in terms of time and cost involved. Earlier works of Hörschler et al. [10] and Zamankhan et al. [11] used a simplified nasal domain and therefore the construction of structured mesh was rendered possible. In an interesting work by Longest and Vinchurkar (2007) [12], several mesh types were compared. The hexahedral mesh was observed to have grid convergence index (GCI) values that were an order of magnitude below the unstructured tetrahedral mesh values for all resolutions considered. This cannot be expected of realistic nasal cavity obtained from actual CT scans. Hence, we find the most of the researchers use unstructured tetrahedral meshing scheme to develop the CFD model. The accuracy of such a mesh is uncertain and therefore its results cannot be utilized to quantify the understanding of flow physiology. Zubair et al. [13] highlighted the need for using hybrid mesh for nasal airflow studies. Recently, hybrid meshes have also been introduced which combines unstructured lower-order internal elements and higher-order pyramid, prism, or hexahedral elements on the surface [12]. Typically they have the advantage of better resolution at near-wall flow field. Lee et al. [14] reportedly used a cluster of prism mesh near the surface to improve the accuracy of the model. But the work did not discuss the usefulness of such a mesh over purely unstructured mesh in the complicated biomedical domain. Some of the popular grid generation softwares Gridgen (Pointwise Inc., USA) and T-grid (Fluent Inc., USA) offer features for developing hybrid mesh.

Hybrid mesh has the ability to resolve the near wall boundary and can be used to develop mesh closer to *y* + = 1. Here *y*+ refers to the nondimensional distance for wall-bounded flow. It is important in turbulence modeling to determine the proper size of the cells near the domain walls. Hybrid mesh is effective in resolving turbulence issues and particularly suitable for use with LES models. In the current study, numerical simulation was carried out to validate the usefulness of hybrid mesh over unstructured tetrahedral mesh. A three-dimensional (3D) nasal cavity model was reconstructed from computed tomographic images (CT) of a healthy Malaysian female. The effect of different mesh type on the fluid flow properties was evaluated.

## 2. Method

**Figure 1:**
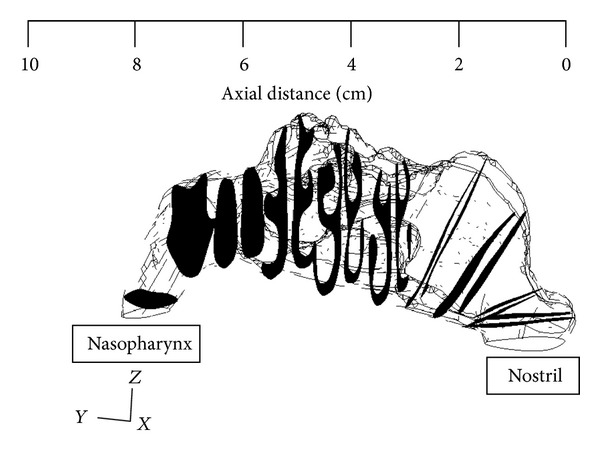
Location of the ten cross-sections along the axial length.

The study was based on an anatomical model of the normal nasal airway obtained from a CT scan of a Malaysian subject from Universiti Sains Malaysia, Medical Campus Hospital. The scan images were segmented slice by slice with an appropriate threshold value using MIMIC (Materialise, Ann Arbor, MI, USA). The 3D polyline data of the nasal cavity was processed in CATIA and meshed with unstructured tetrahedral elements using GAMBIT 2.3.16 (Fluent Inc., Lebanon). The developed 3D nasal cavity model with 10 cross-sectional plane is as shown in Figure 1. These cross-sectional planes represented different locations spanning the entire nasal cavity length and were utilized to extract information about the flow physics inside the flow domain.

**Figure 2:**
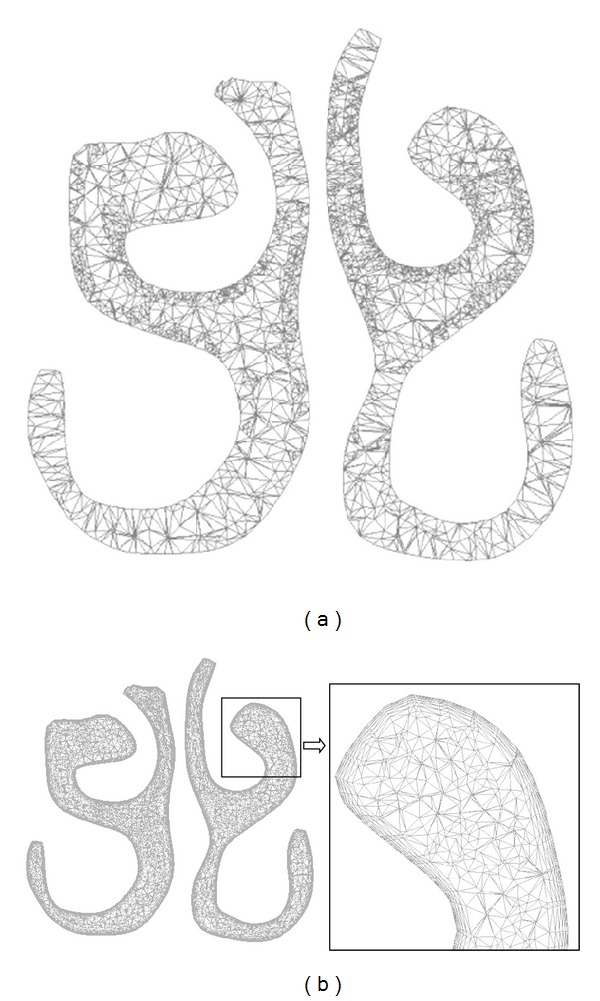
Cross-section of the nasal cavity at a distance of 4.5 cm from the nostril: (a) unstructured mesh and (b) hybrid mesh.

**Figure 3:**
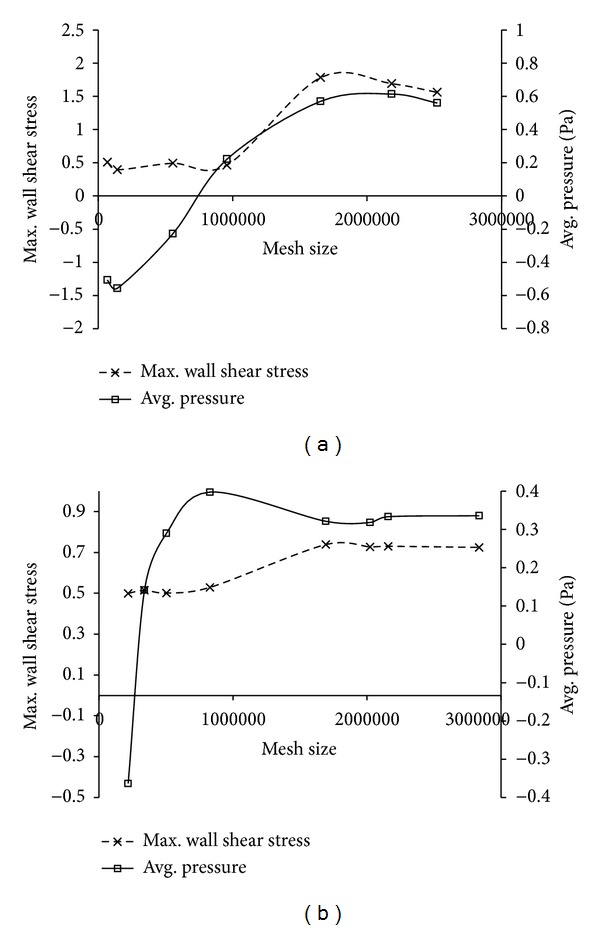
Grid independency study: (a) unstructured mesh and (b) prism mesh.

The two different types of mesh are as depicted in Figure 2.  Figure 2(a) represents the grid display at a location 3.5 cm from the nostril for unstructured mesh type. And Figure 2(b) shows the hybrid mesh at the same location consisting of prism elements stacked at the wall boundary. An unstructured tetrahedral mesh with 1,653,469 elements was developed from the grid independency test (refer Figure 3(a)). This was further adapted using the *y*+ adaptation technique which resulted in a mesh count of 2,522,274 elements. The best possible *y*+ value that could be obtained for this mesh was around 1.31. Also, a hybrid mesh with 1,691,940 elements consisting of a combination of 6 layers of prism cells near the near wall boundary and the tetrahedral elements at the remaining flow domain was obtained from grid independency study (see Figure 3(b)). An initial thickness of 3.8 × 10^−5^ m was maintained for the prism cell to obtain a *y* + <1. The worst cell had the maximum value of skewness of about 0.86. 

The nondimensional wall distance *y*+ is given by

(1) $$\begin{array}{c} y+=\frac{u_{t}y}{\vartheta }, \end{array}$$

where *u*_*t*_ is the skin friction velocity, *y* is the initial height above the wall, and *ϑ* is the kinematic viscosity of air.

To solve the governing mass and momentum conservation equations in each of the mesh style, the CFD package Fluent 6.3.26 (Fluent Inc., Lebanon, PA, USA) has been employed. This commercial software provides an unstructured control-volume-based solution method for both unstructured tetrahedral and hybrid mesh types. The airflow was assumed to be laminar for flow rates up to 15 L/min, and beyond 15 L/min flow was considered turbulent, as predicted by Wen et al., 2008 [1], and Segal et al., 2008 [3]. For turbulence flow, we used the SST *k*-*ω* turbulence model, a two-equation turbulence model, the suitability of which has been explored by Wen et al., 2008 [1], and Mylavarapu et al. [4]. The flow boundary conditions used were as follows: (1) the nasal wall was rigid, (2) the effect of mucus was negligibly small, (3) no-slip condition at the airway wall, and (4) nasal cavity developed was without sinuses, which was commensurate with several earlier studies which neglected the effect of sinus on main flow. The mass flow inlet boundary is defined at the nostril inlet and outflow boundary condition is selected at the outlet. 

### 2.1. Governing Equations of Flow

In the present study steady RANS equations for turbulent incompressible fluid flow with constant properties are used. The governing flow field equations are the continuity and the Reynolds averaged Navier-Stokes equations, which are given by

(2) $$\begin{array}{c} \frac{\partial u_{i}}{\partial x_{j}}=0,\\ \frac{\partial u_{i}u_{j}}{\partial x_{j}}=-\frac{1}{p}\frac{\partial p}{\partial x_{j}}+\frac{\partial }{\partial x_{j}}\left(\vartheta S_{ij}-\overset{-}{u_{ı}^{\prime }u_{ȷ}^{\prime }}\right), \end{array}$$

where *S*_*ij*_ is the main strain rate and calculated by

(3) $$\begin{array}{c} S_{ij}=\frac{1}{2}\left(\frac{\partial u_{i}}{\partial x_{j}}+\frac{\partial u_{i}}{\partial x_{i}}\right), \end{array}$$

and $\overset{-}{u_{ı}^{\prime }u_{ȷ}^{\prime }}=\tau_{ij}$ is the unknown turbulent or Reynolds-stress tensor and *u*_*i*_ represents the velocity fluctuation in *i*-direction. These equations are not a closed set and turbulence models are used to model the turbulent or Reynolds-stress tensor. Shear stress transport SST *k*-*ω* turbulent model closure equations are provided in the work of Menter [15].

Discretization errors arise from numerical algorithms, the mesh style and quality used to discretize the equations, and boundary conditions and is the difference between the exact solution of the governing equations and the discretized system. In this work, the Richardson's extrapolation method has been utilized to determine the mesh-related discretization errors. Celik et al. (2008) [16] and Longest and Vinchurkar (2007) [12] have presented the procedures to apply the Richardson's extrapolation (RE) method to determine the discretization error. Local and global orders of accuracy, extrapolated results, percent errors, and grid convergence indexes are calculated to ensure that a high-fidelity results has indeed been obtained. In total, 3 meshes listed in Table 1 are evaluated to determine the GCI values for each of the mesh type. 

For grid sizes *h*_1_ < *h*_2_ < *h*_3_, the local apparent order of accuracy, *p*, of the simulation was calculated with the following expressions, and the results are tabulated in Table 1. Here the grid refinement factor *r*_21_ = *h*_2_/*h*_1_ and *r*_32_ = *h*_3_/*h*_2_ were maintained greater than 1.3:

(4) $$\begin{array}{c} p=\frac{1}{\ln r_{21}}\left|\ln\left|\frac{\varepsilon_{32}}{\varepsilon_{21}}\right|+q\left(p\right)\right|,\\ q\left(p\right)=\ln\frac{\left(r_{21}^{p}-s\right)}{\left(r_{32}^{p}-s\right)},\\ s=\mathrm{sign}\left(\frac{\varepsilon_{32}}{\varepsilon_{21}}\right), \end{array}$$

where

(5) $$\begin{array}{c} \varepsilon_{21}=\varphi_{2}-\varphi_{1},\\ \varepsilon_{32}=\varphi_{3}-\varphi_{2}. \end{array}$$

Equations (4) are solved using an iterative procedure with an initial guess of *φ*_1_, where *φ*_*n*_ represents the result of the associated grid *n*. 

The extrapolated values *φ*_ext_^21^  and *φ*_ext_^32^ are calculated using the following equations:

(6) $$\begin{array}{c} \varphi_{\text{ext}}^{21}=\frac{\left(r_{21}^{p}\varphi_{1}-\varphi_{2}\right)}{\left(r_{21}^{p}-1\right)},\\ \varphi_{\text{ext}}^{32}=\frac{\left(r_{32}^{p}\varphi_{2}-\varphi_{3}\right)}{\left(r_{32}^{p}-1\right)}. \end{array}$$

The relative errors, *e*_*a*_^21^ and *e*_ext_^21^, are calculated by the expressions:

(7) $$\begin{array}{c} e_{a}^{21}=\left|\frac{\varphi_{1}-\varphi_{2}}{\varphi_{1}}\right|, \end{array}$$

(8) $$\begin{array}{c} e_{\text{ext}}^{21}=\left|\frac{\varphi_{\text{ext}}^{21}-\varphi_{1}}{\varphi_{\text{ext}}^{21}}\right|. \end{array}$$

Finally, the grid convergence index for the most refined mesh is calculated using the expression:

(9) $$\begin{array}{c} {\text{GCI}}_{\text{fine}}^{21}=F_{s}\frac{e_{a}^{21}}{r_{21}^{p_{\text{avg}}}-1}. \end{array}$$

In (8), *F*_*s*_ coefficient serves as a “buffer coefficient” for the extrapolated error approximation GCI and its value for more refined grid cases as in the case of this study is taken as 1.25.

The investigated parameters in the sensitivity study are the maximum velocity magnitude at the nasal valve region and are performed for the case of 20 L/min. Grid convergence indices (GCI) are presented as a percent and can effectively be interpreted as the percent error of the simulation result based on grids analyzed. 

## 3. Results and Discussion

**Figure 4:**
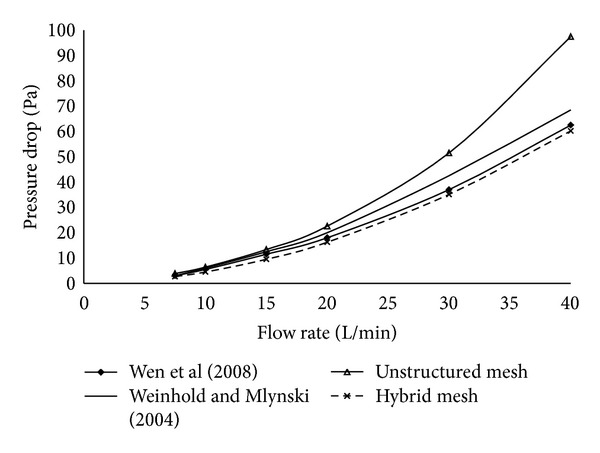
Resistance comparison for hybrid and unstructured mesh types.

The pressure drop obtained using the unstructured computational grid was around 22.6 Pa for a flow rate of 20 L/min, whereas a value of 17.8 Pa was determined for hybrid mesh. The pure unstructured mesh overpredicted the value when compared to that obtained by Wen et al. [1] and Weinhold and Mlynski [17] (18 Pa and 20 Pa, resp.). The resistance obtained varied from 0.026 to 0.124 Pa·sec/mL for flow rate of 7.5 L/min to 40 L/min, respectively. For a flow rate of 15 L/min, the flow resistance obtained was 0.048 Pa·sec/mL. Garcia et al. [18] reported identical results in the range of 0.039 and 0.082 Pa·s/L for a flow rate of 15 L/min.  Figure 4 presents results of hybrid mesh which were similar to that reported in literature. About 21% difference in resistance was obtained between the purely unstructured mesh and the hybrid mesh. In case of the nasal valve, located at a distance of around 2 cm from the anterior region, the maximum velocity obtained with unstructured grid was 4.18 m/s and that with hybrid mesh was 4.76 m/s. On the contrary, 4.82 m/s and 3.1 m/s were reported by Xiong et al. [19] and Croce et al. [20], respectively, for the same location. Thus, considerable differences were observed in the values obtained with respect to hybrid mesh and that of pure unstructured mesh. 

**Figure 5:**
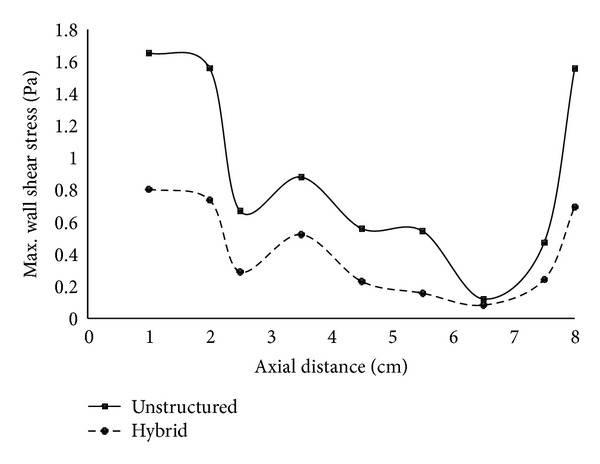
Maximum wall shear stress along the length of the nasal cavity.

A wall shear stress distribution has been plotted for different cross-sections of the nasal cavity.  Figure 5 clearly shows the variation in the maximum wall shear stress obtained for the two types of mesh used. The unstructured mesh is generally not very effective in resolving boundary layer phenomenon and as expected overpredicted the formation of wall shear stress. The anterior and the posterior regions registered more variations. Thus, it can be concluded that hybrid mesh which has a better mesh distribution at the boundary surface is useful in capturing the wall shear stresses.

**Figure 6:**
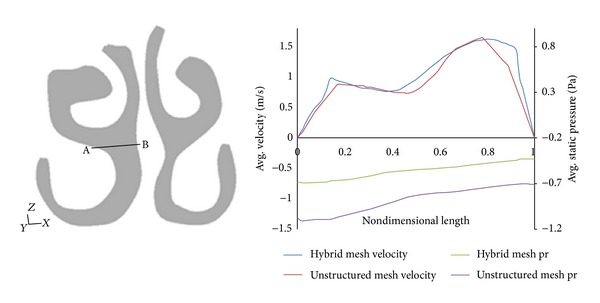
Velocity and pressure distribution along the line AB at a distance of 4.5 cm from the nostril.

**Figure 7:**
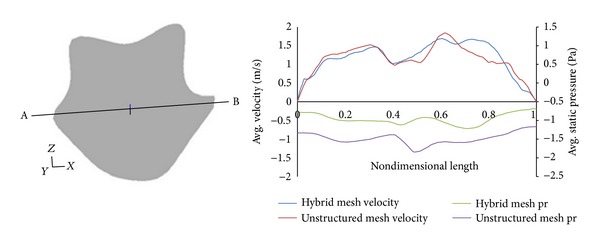
Velocity and pressure distribution along the line AB at the nasopharynx section.

Figures 6 and 7 show the distribution of velocity and pressure at two different locations inside the nasal cavity. These plots have been obtained along the marked line AB as shown in these figures. There is a considerable difference in the values obtained between pure unstructured tetrahedral mesh and the hybrid mesh. The unstructured mesh overpredicts the pressure values, and, moreover, the values of average static velocity in both locations were much lower for hybrid mesh when compared to the unstructured mesh. Therefore, the results obtained using the purely unstructured mesh are not suitable for quantification of flow depicting the nasal physiological function. 

Table 1 presents the comparison for discretisation error measurement. The values have been estimated at the critical location of nasal valve region. Nasal valve is the narrowest part of the nasal cavity and has significant influence on the flow parameters. Unstructured mesh and hybrid mesh behave differently and therefore have different GCI values. The relative error obtained in case of hybrid mesh was only about 2.34%, whereas that determined for unstructured mesh was as high as 12.38%. Moreover, the GCI value for hybrid mesh was ideally less than 1%, whereas for unstructured mesh it was about 3.75%. The unstructured mesh has randomly oriented tetrahedral faces, which are not in tandem with direction of flow. The prism mesh adopted at the near wall boundary provides the necessary alignment to the flow direction, thereby reducing the numerical diffusion errors in case of hybrid mesh scheme. It is probably the reason why tetrahedral mesh has higher GCI values when compared to hybrid mesh type. In context of nasal cavity, one must realize that the nasal architecture is very complicated and is not a uniform pathway. It is a narrow tunnel lined with turbinates and mucous layer, which makes building mesh very difficult. Therefore, it is subjected to very high wall bounded flows and resolving wall layer with appropriate mesh is therefore very important. Thus the hybrid mesh is effective in reducing the diffusion errors at rugged wall boundary and prevents its dissipation into main flow. Earlier studies on bifurcating airways have reported GCI value of about 5% for unstructured mesh schemes [12]. The probable reason for reduced GCI values for unstructured mesh in this study (<5%) is due to high density of mesh cluster at the boundary wall. The *y*+ of about 1.37 was reported for unstructured grid in this study. This clearly shows that mesh resolution at the corrugated nasal walls has significant importance in overcoming errors due to diffusion. However, developing dense mesh along the wall surface using the unstructured tetrahedral type mesh will add to the increased mesh count and thereby is expensive and would take considerably longer duration to solve the equations. On the other hand, hybrid mesh provides a degree of control over mesh resolution in wall boundaries, and mesh with *y* + = 1 is easily generated. Therefore, hybrid mesh has several advantages over a purely tetrahedral mesh type. Moreover, the SST *k*-*ω* turbulence model employed in this work may also contribute to the lower GCI values. SST *k*-*ω* turbulence models were reported to be very useful in wall-bounded flows and have successfully been adopted in many studies in the past [21, 22]. However, further studies are required to authenticate the choice of turbulence models in concluding the lower GCI values for both types of meshes used. Nevertheless, hybrid mesh owing to their ease of development and boundary layer resolution is compatible with complicated geometrical domains such as nasal cavity.

Most of the earlier researchers have employed unstructured mesh in order to evaluate the flow physics inside the complicated nasal domain. It is difficult and time consuming to develop structured meshes. In the absence of structured meshes, hybrid mesh has several advantages over purely unstructured mesh. Hybrid mesh gives better resolution of boundary layer phenomenon. It is particularly useful if one is considering precision turbulence models like the large eddy simulation (LES) models which require well-refined meshes at the wall boundaries [14]. The current study has demonstrated that pure unstructured meshes are not sufficient to resolve the flow features inside the nasal cavity and hence hybrid mesh should be considered in all future nasal flow studies. 

## 4. Conclusion

The usefulness of hybrid mesh over unstructured mesh has been quantified. There is considerable difference in the values of properties that are obtained using unstructured tetrahedral mesh and that of hybrid mesh. Hybrid mesh is easy to develop when compared to pure structural mesh providing good control over the mesh density and is considered useful in resolving wall bounded flows. The complicated anatomy of the nasal cavity makes it difficult to develop structured meshes, and since the unstructured mesh is not accurate enough to capture the flow physics, hybrid mesh offers the best alternative. 

## Figures and Tables

**Table 1:** Comparison of discretisation error measurement for hybrid and unstructured mesh type.

	*φ* = max. velocity (m/s) at the nasal valve (hybrid mesh)	φ = max. velocity (m/s) at the nasal valve (unstructured mesh)
*M*1, *M*2, *M*3	1691940, 504259, 218262	2022162, 450233, 136678
*r*_21_	1.5	1.62
*r*_32_	1.33	1.49
*φ*_1_	4.6576	4.0123
*φ*_2_	4.5487	4.5089
*φ*_3_	4.1892	4.5549
*P*	4.03	4.7830
*φ*_ext_^21^	4.684	3.8919
*e*_*a*_^21^	2.34%	12.38%
*e*_ext_^21^	0.56%	3.09%
GCI^21^_fine_	0.71%	3.75%

## References

[1] Wen J, Inthavong K, Tu J, Wang S (2008). Numerical simulations for detailed airflow dynamics in a human nasal cavity. *Respiratory Physiology and Neurobiology*.

[2] Cheng YS, Yeh HC, Guilmette RA, Simpson SQ, Cheng KH, Swift DL (1996). Nasal deposition of ultrafine particles in human volunteers and its relationship to airway geometry. *Aerosol Science and Technology*.

[3] Segal RA, Kepler GM, Kimbell JS (2008). Effects of differences in nasal anatomy on airflow distribution: a comparison of four individuals at rest. *Annals of Biomedical Engineering*.

[4] Mylavarapu G, Murugappan S, Mihaescu M, Kalra M, Khosla S, Gutmark E (2009). Validation of computational fluid dynamics methodology used for human upper airway flow simulations. *Journal of Biomechanics*.

[5] Riazuddin VN, Zubair M, Abdullah MZ (2011). Numerical study of inspiratory and expiratory flow in a human nasal cavity. *Journal of Medical and Biological Engineering*.

[6] Kleven M, Melaaen MC, Djupesland PG (2012). Computational fluid dynamics (CFD) applied in the drug delivery design process to the nasal passages: a review. *Journal of Mechanics in Medicine and Biology*.

[7] Nagels MA, Cater JE (2009). Large eddy simulation of high frequency oscillating flow in an asymmetric branching airway model. *Medical Engineering and Physics*.

[8] Mihaescu M, Murugappan S, Kalra M, Khosla S, Gutmark E (2008). Large Eddy simulation and Reynolds-Averaged Navier-Stokes modeling of flow in a realistic pharyngeal airway model: an investigation of obstructive sleep apnea. *Journal of Biomechanics*.

[9] Vinchurkar S, Longest PW (2008). Evaluation of hexahedral, prismatic and hybrid mesh styles for simulating respiratory aerosol dynamics. *Computers and Fluids*.

[10] Hörschler I, Brücker Ch, Schröder W, Meinke M (2006). Investigation of the impact of the geometry on the nose flow. *European Journal of Mechanics B*.

[11] Zamankhan P, Ahmadi G, Wang Z (2006). Airflow and deposition of nano-particles in a human nasal cavity. *Aerosol Science and Technology*.

[12] Longest PW, Vinchurkar S (2007). Effects of mesh style and grid convergence on particle deposition in bifurcating airway models with comparisons to experimental data. *Medical Engineering and Physics*.

[13] Zubair M, Abdullah MZ, Suzina AH, Rushdan I, Shuaib IL, Ahmad KA (2012). A critical overview of CFD modeling of nasal airflow. *Journal of Medical and Biological Engineering*.

[14] Lee JH, Na Y, Kim SK, Chung SK (2010). Unsteady flow characteristics through a human nasal airway. *Respiratory Physiology & Neurobiology*.

[15] Menter FR (1994). Two-equation eddy-viscosity turbulence models for engineering applications. *AIAA journal*.

[16] Celik IB, Ghia U, Roache PJ, Freitas CJ, Coleman H, Raad PE (2008). Procedure for estimation and reporting of uncertainty due to discretization in CFD applications. *ASME Journal of Fluids Engineering*.

[17] Weinhold I, Mlynski G (2004). Numerical simulation of airflow in the human nose. *European Archives of Oto-Rhino-Laryngology*.

[18] Garcia GJM, Bailie N, Martins DA, Kimbell JS (2007). Atrophic rhinitis: a CFD study of air conditioning in the nasal cavity. *Journal of Applied Physiology*.

[19] Xiong G, Zhan J, Zuo K, Li J, Rong L, Xu G (2008). Numerical flow simulation in the post-endoscopic sinus surgery nasal cavity. *Medical and Biological Engineering and Computing*.

[20] Croce C, Fodil R, Durand M (2006). In vitro experiments and numerical simulations of airflow in realistic nasal airway geometry. *Annals of Biomedical Engineering*.

[21] Zhen TK, Zubair M, Ahmad KA (2011). Experimental and numerical investigation of the effects of passive vortex generators on Aludra UAV performance. *Chinese Journal of Aeronautics*.

[22] Long AK, Zubair M, Ahmad KA (2011). A static thrust measurement of two types of perforated solid propellant grain configurations. *Journal of Aerospace Engineering*.

